# Supplementary material for: Intramedullary Nailing with and without the Use of Bone Cement for Impending and Pathologic Fractures of the Humerus in Multiple Myeloma and Metastatic Disease
Source: Cancers (Basel). 2023 Jul 13;15(14):3601. doi: 10.3390/cancers15143601 (PMC10377631; doi:10.3390/cancers15143601)
Supplement: Supplementary file 1 [file cancers-15-03601-s001.zip › Supplement S1.pdf]

cancers

MDPI

Intramedullary Nailing with and without the Use of Bone Cement for Impending and Pathologic Fractures of the Humerus in Multiple Myeloma and Metastatic Disease

Andriy Kobryn, Patrick Nian, Joydeep Baidya, Tai L. Li and Aditya V. Maheshwari

Supplement S1. Literature review of the last 30 years summarizing complication rates and mean survivorship in patients undergoing intramedullary nailing for metastatic humeral disease.

Reference	Operative Modality*	Total Nails	Cemented Nails (Cement only in the tumor cavity)§	Cemented Nails (filling of entire intramedullary Canal with cement)§	Uncemented Nails§	Complication Rate N (%)§	Surgical Complication Rate N (%)§	Medical and Systemic Complication Rate N (%)§	Mean Survival (Months)§	Functional Scores
Ingman et al., 1994 [25]	IMN	15	NR	NR	15	5 (33.3%)	4 (26.7%)	1 (7%)	3	NR
Dijkstra et al., 1996 [5]	IMN<br>PSF	18	NR	NR	18	7 (39.9%)	4 (22.2%)	3 (16.7%)	5	NR
Redmond et al., 1996 [30]	IMN	16	NR	NR	16	1 (6.3%)	1 (6.3%)	0 (0%)	4	NR
Vandeweyer et al., 1997 [34]	IMN	18	NR	18	NR	1 (5.6%)	1 (5.6%)	NR	NR	Enneking [45]: 93.75%
Tome et al., 1998 [33]	IMN	14	NR	NR	14	4 (28.6%)	4 (28.6%)	NR	12	NR
Rommens et al., 1998 [31]	IMN	190	NR	NR	190	21 (11.1%)	21 (11.1%)	NR	NR	NR
Gebhart et al., 2001 [35]	IMN<br>PSF<br>EPR	38	NR	38	NR	1 (2.6%)	1 (2.6%)	NR	9	NR
Bauze et al., 2003 [24]	IMN	31	4	NR	27	7 (22.6%)	7 (22.6%)<br>Cement augmented: 2 (6.5%)<br>Uncemented: 5 (16.1%)	NR	6	NR
Bickels et al., 2005 [36]	IMN<br>EPR	39	NR	39	NR	NR	NR	NR	NR	Postop MSTS: > 68%Ψ
Hunt et al., 2006 [16]	IMN	11	NR	6	5	2 (18.2%)	2 (18.2%)<br>Cemented: 1 (9.1%)<br>Uncemented: 1 (9.1%)	NR	NR	NR
Thai et al., 2006 [14]	IMN<br>EPR	51	NR	44	7	3 (5.9%)	3 (5.9%)<br>Cemented: 1 (2.0%)<br>Uncemented: 2 (3.9%)	NR	32	NR
Atesok et al., 2007 [20]	IMN	24	5	NR	19	3 (12.5%)	3 (12.5%)<br>Cement augmented: 0 (0 %)<br>Uncemented: 3 (12.5%)	NR	NR	NR
Camnasio et al., 2008 [11]	IMN<br>EPR	40	40	NR	NR	NR	NR	NR	26	NR
Ofluoglu et al., 2009 [28]	IMN	24	NR	NR	24	2 (8.3%)	2 (8.3%)	0 (0%)	NR	Postop MSTS: 64%
Sarahrudi et al., 2009 [4]	IMN<br>PSF	19	NR	NR	19	2 (10.5%)	2 (10.5%)	0 (0%)	4	NR
Spencer et al., 2010 [32]	IMN	37	NR	NR	37	1 (3.2%)	1 (3.2%)	0 (0%)	7	NR
Piccioli et al., 2010 [8]	IMN<br>EPR	57	NR	48	9	13 (22.8%)	13 (22.8%)<br>Cemented: NR<br>Uncemented: NR	NR	8	Baseline/3/8 months postop MSTS: 67.8/72.7/79.2
Pretell et al., 2010 [18]	IMN	23	NR	NR	23	6 (26%)	0 (0%)	6 (26%)	23	Preop/postop VAS pain: 89.5/14.5
Laitinen et al., 2011 [2]	IMN	40	NR	21	19	7 (17.5%)	7 (17.5%)<br>Cemented: 4 (10.0%)	NR	15	1 week/6 week/6 months postop MSTS (cemented/uncemented): (63.0/39.7%†),

							Uncemented: 3 (7.5%)			(66.8/52.7%†), (70.5/67.8%)
Kim et al. 2011 [37]	IMN	15	NR	15	NR	0 (0%)	0 (0%)	0 (0%)	9	Preop/postop MSTS: 10.6/19.9
Wedin et al., 2012 [15]	IMN<br>EPR<br>PSF	148	45	NR	81	13 (8.8%)	13 (8.8%)<br>Cement augmented:4 (2.7%)<br>Uncemented: 9 (6.1%)	NR	NR	Median Karnofsky: 70Ψ
Chen et al., 2014 [38]	IMN	8	8	NR	NR	0 (0%)	0 (0%)	0 (0%)	12	Preop/postop VAS pain: 92.5/13.75<br>Constant-Murley: 71.4<br>ASES: 82.5<br>Mayo Elbow Performance: 79.4°
Choi et al., 2016 [21]	IMN	32	NR	32	NR	1 (3.1%)	1 (3.1%)	NR	14	Preop/1 months postop VAS pain: 8.3/2.1<br>3 months postop MSTS: 27.7<br>3 months postop Karnofsky: 75.6
Janssen et al., 2016 [17]	IMN<br>EPR<br>PSF	189	1	6	182	28 (14.8%)	10 (5.3%)<br>Cement augmented: NR<br>Cemented: NR<br>Uncemented: NR	18 (9.5%)<br>Cement augmented: NR<br>Cemented: NR<br>Uncemented: NR	NR	NR
Kim et al., 2016 [26]	IMN	70	43	NR	27	8 (11.9%)	4 (5.9%)<br>Cement augmented: NR<br>Uncemented: NR	4 (5.9%)<br>Cement augmented: NR<br>Uncemented: NR	10	Preop/immediate postop/follow up postop VAS pain (cemented/uncemented): (9.5/9.5), (3.8/6.0†), (3.3/6.6†)
Park et al., 2018 [39]	IMN	23	23	NR	NR	3 (13.0%)	0 (0%)	3 (13.0%)	NR	Preop/1 week postop/6 weeks VAS pain: 8.2/4.9/2.9
Moura et al., 2019 [27]	IMN	86	NR	NR	86	4 (4.7%)	4 (4.7%)	NR	10	Preop/postop MSTS score: 26.0/72.6%
Park et al., 2019 [40]	IMN	4	NR	4	NR	0 (0%)	0 (0%)	0 (0%)	12	Preop/1 week postop/4 weeks postop VAS pain: 7.3/3.3/2.3<br>Preop/4 weeks postop MSTS: 14/26
Pizzo et al., 2020 [29]	IMN	26	NR	26	NR	2 (7.7%)	2 (7.7%)	0 (0%)	NR	Preop/postop MSTS: 10.5/26.1
Ricard et al., 2021 [41]	IMN<br>PSF	8	NR	2	6	2 (25.0%)	1 (12.5%)	1 (12.5%)	NR	Baseline/2 week/6 week/12 week/26 week/52 weeks postop MSTS: 20/66/72/82/85/100<br>Baseline/2 week/6 week/12 week/26 week/52 weeks postop Toronto Extremity Salvage Score (TESS): 35/47/71/70/69/98
Current Study	IMN	100	Not done exclusively	53	47	21 (21.0%)	8 (8.0%)<br>Cemented: 6 (6.0%)<br>Uncemented: 2 (2.0%)	14 (14.0%)<br>Cemented:7 (7.0%)<br>Uncemented: 7 (7.0%)	10	Preop/postop MSTS score (cemented/uncemented): (40.2/66.7†), (89.8/90.9)
