# Supplementary material for: Intramedullary Nailing with and without the Use of Bone Cement for Impending and Pathologic Fractures of the Humerus in Multiple Myeloma and Metastatic Disease
Source: Cancers (Basel). 2023 Jul 13;15(14):3601. doi: 10.3390/cancers15143601 (PMC10377631; doi:10.3390/cancers15143601)
Supplement: Supplementary file 1 [file cancers-15-03601-s001.zip › Supplement S2.pdf]

cancers

MDPI

Intramedullary Nailing with and without the Use of Bone Cement for Impending and Pathologic Fractures of the Humerus in Multiple Myeloma and Metastatic Disease

Andriy Kobryn, Patrick Nian, Joydeep Baidya, Tai L. Li and Aditya V. Maheshwari

1. Surgical Technique

Surgeries were performed under general anesthesia with or without scalene block, using an antegrade Stryker T2 proximal humerus nail (Stryker Trauma GmbH, Schönkirchen, Germany) [54]. Patients were positioned supine (95 [96.0%] cases) with a bump under their ipsilateral scapula on a radiolucent table or in a beach chair (3 [3.0%] cases) due to the possibility of an arthroplasty procedure, or laterally (1 [1.0%] case) for other concomitant surgeries. Fluoroscopic images were obtained before prepping, with the arm in adduction and external rotation to ensure that visualization of the entire bone was possible, and the junction between the articular surface and the greater tuberosity (the entry point) was seen on an antero-posterior (AP) view. Lateral views were obtained by rotating the arm as one piece.

1.1. Uncemented Nailing

A 2-3 cm incision (with arm in external rotation as above) was made starting anterolateral down from the acromion in line with the deltoid fibers which are split along the incision, taking care not to injure the axillary nerve and posterior humeral circumflex vessels distally. The subdeltoid bursa and rotator cuff was also incised and tagged, exposing the capsule of the shoulder joint. The humeral head was exposed after incising the capsule. The entry point was made medial to the greater tuberosity using a curved awl and/or a guide-wire, just medial to the junction of the articular and non-articular surface, and confirmed using biplanar fluoroscopy to be aligned to the shaft. For proximal humeral lesions, we erred more on the medial site to minimize lateral cut out by the reamer and/or nail. This was either followed by biopsy (if needed) and placement of a long-beaded guide wire. Proximal reaming (10 mm) was done to the level of metaphysis as per manufacturer recommendation. No further reaming was performed unless the shaft was too narrow. Tumors located within the proximal third of the humerus were debulked using curettes, rongeurs, and reamers as much as possible to reduce their burden and aid in cement placement. The beaded guide wire was exchanged to a non-beaded wire, and the 8 mm nail was placed, and locked proximally with 2 to 3 interlocking screws through the provided jig. While inserting the nail, it was aligned for the distal AP screw-hole to be placed anteriorly, just lateral to the biceps tendon to minimize injury to neurovascular structures. Depending on the stability, 1 (rarely 2) distal anteroposterior interlocking screw was placed using a free hand perfect circle method while protecting the neurovascular structures. The wounds were lavaged and closed in layers including direct repair of the capsule, rotator cuff and deltoid, followed by subcutaneous tissue and skin. No drain was placed.

1.2. Cemented Nailing

For cemented nails, the technique was modified as following. The skin incision was 2 to 3 cm longer (4 to 6 cm) to help with removal of excess cement at the end of the procedure and to ensure no extravasation in the joint. After initial steps as above till tumor debulking, flexible reamers were used to open the canal to the isthmus (often to a size of 11 to 12 mm) and accommodate the cement gun nozzle, which was thinner (10 mm) and longer (300 mm) than the usual nozzle used for hip arthroplasty (Stryker Instruments,

Kalamazoo, MI, USA). Care was taken not to perforate the bone distally with guidewire or the cement can extrude distally, especially if the bone quality was poor. Once appropriate length of the nail was chosen, it was assembled and was placed inside the canal to ensure its passage and position over a non-beaded guide wire and checked with biplanar fluoroscopy. The impactor was removed and the guidewire was cut appropriately short (about 2-3 inches from the jig) to allow the passage of the humerus cement nozzle over it to appropriate depth in the canal, but not too short to prevent its extraction later on. The nail was then removed but the guidewire was kept in. The cement gun nozzle was then test-placed in the canal over the guidewire to ensure proper placement and to get an idea about the direction. The debris inside the canal was removed with suction as much as possible.

Two to three packets of medium viscosity bone cement (Simplex P with 1.2 gm of Tobramycin, Stryker, Mahwah, NJ) were mixed under vacuum and placed in a standard cement gun but different nozzle as above. The locking mechanism of the nozzle to the cement gun was ensured. Because of the longer and thinner cement nozzle, the cement was used in a more liquid sticky state in contrast to the usual 'tooth-paste' consistency in the femur. A cotton-gauge can be placed around the bone hole to minimize cement extrusion in the joint or surrounding tissues. The nozzle was placed over the previously cut guide-wire in appropriate direction as determined earlier and inserted till the isthmus or reamed area. The cement was injected in the canal in 'fill mode' without much pressurization in a retrograde manner (distal to proximal). In cases of a fracture, to minimize cement extrusion at the fracture site, either a manual hand pressure or a tourniquet was applied. If there is a large tumor cavity, then more cement is injected in that area under fluoroscopic guidance. The cement gun was then withdrawn and the preassembled nail (which can be coated with cement too) was gently placed in the canal over the guide wire. The reduction was held appropriately in cases of pathologic fractures. Usually, the nail went in without much difficulty as the canal had been over-reamed. The guide wire was quicky removed and an impactor was placed over the jig. The nail was then tapped to appropriate position as confirmed under fluoroscopy to avoid impinging the rotator cuff tendons or the acromion. Excessive cement was removed. The reduction and nail were held in place until the cement was cured. Once cement was cured, excessive cement was removed. The gauge was also removed and often it pulled out cured cement pieces that were stuck to the gauge. Fluoroscopy was used to look for any other loose cement pieces and they were removed. Two to three proximal interlocking screws were placed via the provided jig using the previous incision or another stab incision. The drill and screw often had to go through the cement and thus extra caution was required at this step. Depending on the stability of the fracture and purchase of the cement, no distal locking was required in most cases. Incomplete filling of cement was not uncommon. Seven (13.5% of 52) cemented nails required one distal locking screw due to unsatisfactory cement filling of the canal/tumor cavity and/or rotational instability despite cementation. Although the fracture often acted as a natural vent site, no formal venting holes were created. Some cement extrusion at fracture site was common but did not require any removal nor created future complications, except one case where the cement broke and became a mobile loose body and was removed in the same setting.

Acknowledgments: The technique of cementation has been adapted from Dr Robert M. Henshaw, MD.
